# Serological evidence indicates widespread distribution of rickettsioses in Myanmar

**DOI:** 10.1016/j.ijid.2020.12.013

**Published:** 2021-02

**Authors:** Philip N.D. Elders, Myo Maung Maung Swe, Aung Pyae Phyo, Alistair R.D. McLean, Htet Naing Lin, Kyaw Soe, Wei Yan Aung Htay, Ampai Tanganuchitcharnchai, Thel K. Hla, Ni Ni Tun, Thin Thin Nwe, Myat Myat Moe, Win May Thein, Ni Ni Zaw, Wai Mon Kyaw, Htun Linn, Yin Yin Htwe, Frank M. Smithuis, Stuart D. Blacksell, Elizabeth A. Ashley

**Affiliations:** a Myanmar Oxford Clinical Research Unit, Yangon, Myanmar; b Centre for Tropical Medicine and Global Health, Nuffield Department of Medicine, University of Oxford, Oxford, United Kingdom; c Mahidol-Oxford Tropical Medicine Research Unit, Faculty of Tropical Medicine, Mahidol University, Bangkok, Thailand; d Medical Action Myanmar, Yangon, Myanmar; e Magway General Hospital and University of Medicine, Magway, Myanmar; f University of Medicine 2, Yangon, Myanmar; g Mandalay General Hospital and University of Medicine, Mandalay, Myanmar; h Monywa General Hospital, Monywa, Myanmar; i National Health Laboratory, Yangon, Myanmar; j Lao-Oxford-Mahosot Hospital-Wellcome Trust Research Unit, Microbiology Laboratory, Mahosot Hospital, Vientiane, Lao Democratic People’s Republic

**Keywords:** Scrub typhus, Murine typhus, Spotted fever group, Rickettsial infections, Seroprevalence, Myanmar

## Abstract

**Background:**

Little research has been published on the prevalence of rickettsial infections in Myanmar. This study determined the seroprevalence of immunoglobulin G (IgG) antibodies to rickettsial species in different regions of Myanmar.

**Methods:**

Seven hundred leftover blood samples from patients of all ages in primary care clinics and hospitals in seven regions of Myanmar were collected. Samples were screened for scrub typhus group (STG), typhus group (TG) and spotted fever group (SFG) IgG antibodies using enzyme-linked immunosorbent assays (ELISA). Immunofluorescence assays were performed for the same rickettsial groups to confirm seropositivity if ELISA optical density ≥0.5.

**Results:**

Overall IgG seroprevalence was 19% [95% confidence interval (CI) 16–22%] for STG, 5% (95% CI 3–7%) for TG and 3% (95% CI: 2–5%) for SFG. The seroprevalence of STG was particularly high in northern and central Myanmar (59% and 19–33%, respectively). Increasing age was associated with higher odds of STG and TG seropositivity [per 10-year increase, adjusted odds ratio estimate 1.68 (*p* < 0.01) and 1.24 (*p* = 0.03), respectively].

**Conclusion:**

Rickettsial infections are widespread in Myanmar, with particularly high seroprevalence of STG IgG antibodies in central and northern regions. Healthcare workers should consider rickettsial infections as common causes of fever in Myanmar.

## Highlights

• Diagnosis of rickettsial infections is difficult in low-resource settings; this leads to delays in receiving appropriate treatment.
• Before this study, the distribution of rickettsioses in Myanmar was not known.
• This serosurvey shows that rickettsioses are widespread in Myanmar.
• Particularly high prevalence of scrub typhus was found in central and northern regions.

## Introduction

Rickettsial infections are among the most important causes of non-malarial fever in Southeast Asia (Acestor et al., 2012, Wangdi et al., 2019). Rickettsial bacteria are obligate intracellular Gram-negative coccobacilli that are transmitted to humans through bites of infected fleas, mites, ticks and lice (Trung et al., 2017). Rickettsioses can be divided into three major groups: the scrub typhus group (STG), the typhus group (TG) and the spotted fever group (SFG). TG and SFG are caused by species within the same genus of *Rickettsia*; TG consists of endemic murine typhus (*Rickettsia typhi*) and epidemic typhus (*Rickettsia prowazekii*), and SFG contains over 20 different species worldwide. STG is caused by the genus *Orientia*, which includes the species *Orientia tsutsugamushi* and the recently described *Candidatus* Orientia chuto and *Candidatus* Orientia chiloensis (Abarca et al., 2020, Izzard et al., 2010). The major causes of rickettsioses in Southeast Asia are *O. tsutsugamushi, R. typhi* and some members of SFG (Aung et al., 2014).

Rickettsial infections typically present with acute fever, often accompanied by headache, myalgia, nausea and vomiting, sometimes with a rash appearing after 3–5 days of illness (Aung et al., 2014, Walker, 2003). Overall mortality of rickettsial infections differs by species and can be as low as 0.4% for murine typhus, but has been reported to be approximately 6% for scrub typhus if left untreated, and higher if patients develop complications such as meningitis or meningoencephalitis (Bonell et al., 2017, Doppler and Newton, 2020, Taylor et al., 2015). These types of central nervous system infections have been found to be caused by rickettsial infections in a significant proportion of patients in Laos (Dittrich et al., 2015). An eschar is a strong predictor of STG and tick-borne SFG rickettsioses, and can differentiate these from murine typhus and other infectious diseases (Aung et al., 2014, Walker, 2003). However, the presence of an eschar varies widely (Saraswati et al., 2018) and its detection requires thorough physical examination, which is often not performed. Other signs and symptoms are non-specific and vary, which makes clinical diagnosis difficult. Diagnosis is further complicated by a lack of reliable point-of-care tests, the organisms not being picked up by routine culture media, and a general lack of standardized and validated assays (Trung et al., 2017).

In order to estimate the burden of rickettsioses in different areas, epidemiological information on their distribution is crucial. A number of studies have tried to estimate the burden of different rickettsioses in Southeast Asia (Aung et al., 2014), but the results are often difficult to compare due to the use of different inclusion and exclusion criteria, and different serological tests. The seroprevalence of rickettsial infections appears to vary substantially between countries (Khan et al., 2016, Maude et al., 2014, Trung et al., 2017, Tshokey et al., 2017). In Myanmar, studies during the Second World War described endemic cases and multiple outbreaks of scrub typhus in Chinese and American troops operating in northern Myanmar (Mackie, 1946, Sayen and Pond, 1946). Recent studies in Myanmar have found that rickettsial infections are very uncommon causes of fever in primary care clinics in Yangon (Althaus et al., 2020), but high STG immunoglobulin M (IgM) and IgG titres have been found in suspected cases of scrub typhus in Sagaing and Magway (Win et al., 2020). Other studies on the Thai side of the border with Myanmar have found convincing evidence of all major groups of rickettsial infections: 4–12% of patients presenting with (non-malarial) fever were diagnosed with rickettsial infections (Ellis et al., 2006; McGready et al., 2010; Pickard et al., 2004; Watthanaworawit et al., 2013). These studies suggest that rickettsioses are likely to be important causes of disease in specific geographical regions of Myanmar. However, no studies have been published on the prevalence of different rickettsial infections in multiple regions of Myanmar. Data on the current burden will enable prioritization of resources to improve recognition of rickettsial infections in the most-affected areas.

The presence of IgG antibodies in the population can be measured to determine the number of people who are likely to have had previous exposure to rickettsial infections. The indirect immunofluorescence assay (IFA) is generally considered to be the gold standard test for serological diagnosis of rickettsial infections (Aung et al., 2014, Blacksell et al., 2016a). However, IFA has several limitations in that it is difficult to standardize due to operator subjectivity, it needs appropriate local diagnostic cut-offs, and requires improvement in terms of standardization and ease of use (Blacksell et al., 2007, Blacksell et al., 2016a, Phetsouvanh et al., 2013). An alternative serological test that is cheaper, easier to use and has been evaluated extensively is the enzyme-linked immunosorbent assay (ELISA) (Blacksell et al., 2016a, Blacksell et al., 2016b, Blacksell et al., 2018, Lim et al., 2015). Recent research has found that an in-house ELISA has high sensitivity to detect IgG IFA titres at levels suitable for seroprevalence studies for both STG and TG [sensitivity of both >95%, (Elders et al., 2020, n.d.)], and could therefore be used as an initial screening test for the presence of IgG antibodies before final confirmation of seropositivity with IFA.

To estimate the burden of rickettsioses in different regions of Myanmar, this seroprevalence study was conducted to test for IgG antibodies against rickettsial infections in leftover blood from patients at seven clinics and hospitals in seven regions of Myanmar, using ELISA as screening with subsequent seropositivity confirmed by IFA.

## Methods

**Figure 1:**
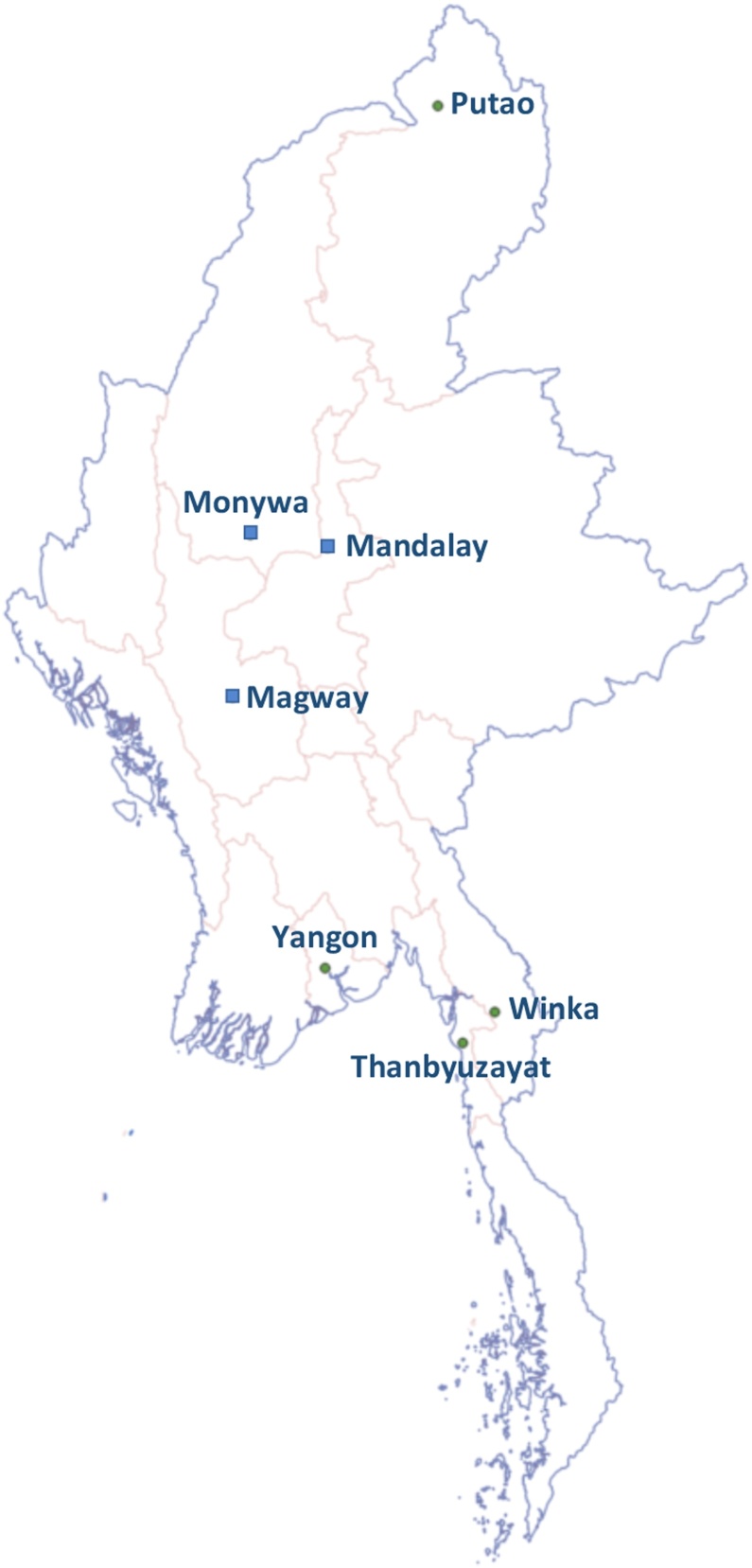
Study sites. Blue squares, government hospitals; green circles, primary care clinics of Medical Action Myanmar, a medical non-governmental organization.

Patients who presented at three Myanmar government hospitals [Monywa General Hospital (Sagaing), Mandalay General Hospital and University of Medicine (Mandalay), and Magway General Hospital (Magway)] and four primary care clinics of Medical Action Myanmar, a medical non-governmental organization [Putao (Kachin), Thanbyuzayat (Mon), Hlaing Thar Yar (Yangon) and Winka (Kayin)], were included in this study (Figure 1). One hundred participants were included from each site, making a total of 700 participants.

All patients who presented to study hospitals or clinics who required a blood draw for any reason after consultation with a medical doctor were included in this study. Written informed consent was provided by all participants or by parents/caregivers if children were under 18 years of age. Participants were excluded if there was less than 500 μl of leftover blood. Participant inclusion started in June 2019 and was completed in October 2019. Enrolment of participants was stratified by age group, following the 2018 United Nations world population prospectus age distribution (UNdata. Explorer.) in the following age groups: 0–<5 years (8%); 5–<18 years (25%), 18–<55 years (54%) and ≥55 years (13%). If insufficient participants were recruited at a site after 3 weeks, age stratification was abandoned for financial and logistical reasons, and sequential participants of any age presenting to the study site were recruited until 100 participants were included. The samples were anonymized, with region, gender and age recorded on data sheets for each site. The samples were stored locally at −20 °C. Samples from 39 participants in Magway were thawed inadvertently during storage at the location, and were replaced with samples from additional participants. Once sample collection was completed at a site, all frozen samples were shipped to the Myanmar Oxford Clinical Research Unit laboratory on dry ice, where they were stored centrally at −80 °C. Once all 700 samples had been collected, they were transported on dry ice from Yangon to Mahidol-Oxford Tropical Medicine Research Unit (MORU) in Bangkok, Thailand for laboratory analysis.

### Laboratory analysis

The seroprevalence of IgG for rickettsial infections was determined to estimate the number of previous infections in the population. All samples were screened for IgG antibodies to STG (*O. tsutsugamushi* strains Karp, Kato, Gilliam and TA716), TG (*R. typhi* strain type Wilmington) and SFG rickettsiae (*Rickettsia honei* and *Rickettsia conorii)* using antigens available from the US Naval Medical Research Center (NMRC) with MORU in-house ELISAs. MORU in-house ELISAs for STG, TG and SFG are based upon the NMRC ELISA protocols using the same method, differing only in antigen composition for each rickettsial infection (Blacksell et al., 2016a, Graf et al., 2008, Khan et al., 2016, Phanichkrivalkosil et al., 2019). U-bottomed 96-well microtitre ELISA plates were prepared by coating half the plate with the NMRC antigens for either STG, TG or SFG by adding antigen diluted in phosphate buffered saline (PBS) 100 μl/well at 1:8000, 1:4000 and 1:2000 dilutions for each rickettsial group, respectively. The other half of the plate served as a control with PBS alone. Coated plates were covered and stored in a moist chamber at 4 °C for 36–48 h. After incubation, the plates were blocked with 200 μl/well of blocking buffer (5% skimmed milk in wash buffer) for 1 h, and then rinsed three times with wash buffer (0.1% Tween 20 in PBS). Serum samples were diluted 1:100 in blocking buffer, added as 100 μl/well, and incubated for 1 h in a moist chamber at room temperature. Plates were washed four times with wash buffer. Subsequently, horseradish-peroxidase-conjugated goat anti-human IgG (Invitrogen Corp., Carlsbad, CA, USA) was added at a 1:1000 dilution in blocking buffer at 100 μl/well, and incubated for 1 h in a moist chamber at room temperature. Following incubation of this secondary antibody, plates were washed four times with wash buffer. Next, tetramethylbenzidine substrate (KPL Inc., Gaithersburg, MD, USA; 100 μl/well) was added and incubated for 15 min at room temperature in the dark. Finally, 100 μl/well of 1 M hydrochloric acid was added to each well to stop the reaction. The plates were read at 450 nm using a microplate reader (Multiskan FC; ThermoFisher Scientific, Waltham, MA, USA) with the optical densities (ODs) from wells without antigen subtracted as background absorbance to generate a final net OD. Negative and positive control samples were used as a control of assay performance, and were included in four wells each on each plate. If a sample had an ELISA IgG OD ≥ 0.5 (Elders et al., 2020, Saraswati et al., 2019), a subsequent IgG IFA was performed for the same rickettsial group, using the same antigens. The IFA determined the IgG antibody titre by serially diluting samples two-fold from 1:100 to 1:25,600, and the endpoint was determined as the highest titre displaying specific fluorescence, as described previously (Paris et al., 2011, Phakhounthong et al., 2020).

### Definition of seropositivity

In this study, seropositivity was defined as IFA IgG titre ≥1:100 after initial screening with an ELISA that had OD ≥ 0.5, indicative of previous exposure to a rickettsial infection (Kim et al., 2010, Phakhounthong et al., 2020).

### Data analysis

Participant characteristics were described using number and percentage for gender and age group, and median and interquartile range (IQR) for age. Fitted linear regression lines were plotted for age in years against ELISA IgG OD. Prevalence and 95% confidence intervals (CI) (using Wilson score intervals) of STG, TG and SFG IgG with ELISA positivity, and with ELISA positivity and subsequent IFA seropositivity were calculated. Logistic regression (unadjusted and adjusted) was used to compute odds ratios (OR) and 95% CI to quantify the association between participant characteristics (gender, age per 10 years and region) and the odds of STG, TG and SFG IgG seropositivity on IFA. All participants with ELISA OD < 0.5 were not tested with IFA, and were considered to be seronegative. The region with the highest number of participants with an IFA titre ≥1:100 for all three rickettsial groups was used as the reference region. Data management and analysis were performed using STATA Version 15.1 (StataCorp, College Station, TX, USA).

This study was approved by the University of Public Health Institutional Review Board, Yangon, Myanmar (UPH-IRB 2019/Research/22) and the Oxford Tropical Research Ethics Committee, Oxford, UK (OxTREC Reference 552−18).

## Results

In total, 700 samples were collected from seven different hospitals and clinics in Myanmar. Half (49.9%) of the participants were male, and the median age was 31 years (IQR 21–45 years, range 3 months–87 years). Participant characteristics are shown by region in Table 1.

**Table 1:** Participant characteristics by region.

Location	*n*	Male gender, %	Median age (IQR), years	% of participants by age group, years			
				0–<5	5–<18	18–<55	≥55
Kachin	100	66	33 (25–45)	3	4	77	16
Sagaing	100	81	20 (11–28)	14	14	66	6
Mandalay	100	37	43 (33–52)	0	2	76	22
Magway	100	46	31 (21–56)	7	6	61	26
Kayin	100	39	28 (19–39)	4	18	66	12
Mon	100	32	29 (15–41)	5	25	56	14
Yangon	100	48	31 (24–40)	3	5	83	9
Total	700	49.9	31 (21–45)	5.1	10.6	69.3	15.0

IQR, interquartile range.

### ELISA screening

**Figure 2:**
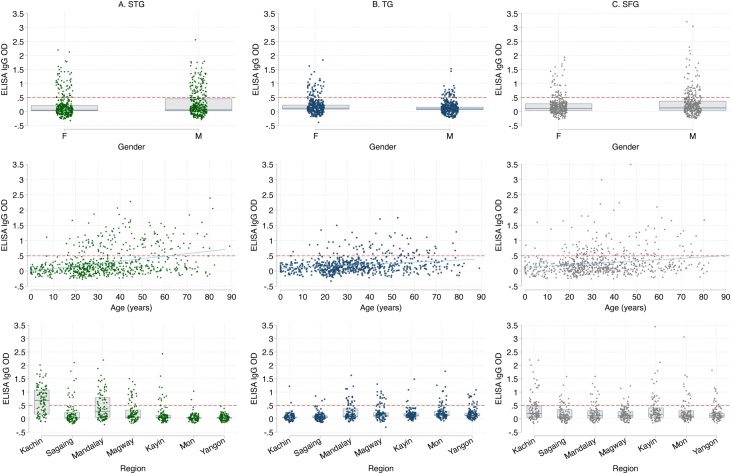
Optical densities (ODs) of enzyme-linked immunosorbent assays (ELISA) for each gender, age in years, and region for scrub typhus group (A), typhus group (B) and spotted fever group (C). Boxes denote quartiles (75th percentile, median, 25th percentile). The red dashed line indicates ELISA OD ≥ 0.5, which was used as the cut-off point for further testing with immunofluorescence assay. The blue line is a fitted linear regression line between age and ELISA IgG OD.

All participants were screened with ELISA for STG, TG and SFG IgG antibodies. The median (IQR) ODs with ELISA are shown in Table S1 (see online supplementary material) and presented in Figure 2. Table S2 (see online supplementary material) shows the percentage of samples with ELISA OD ≥ 0.5 with 95% CI. The overall percentages of ELISA OD ≥ 0.5 for STG, TG and SFG were 20.1% (95% CI 17.3–23.3%), 10.1% (95% CI 8.1–12.6%) and 14.4% (95% CI 12.0–17.2%), respectively. There were 15 participants with ELISA OD ≥ 0.5 for both STG and TG, 39 participants with ELISA OD ≥ 0.5 for STG and SFG, 10 participants with ELISA OD ≥ 0.5 for TG and SFG, and two participants with ELISA OD ≥ 0.5 for all three groups. Kachin had the highest percentage with ELISA OD ≥ 0.5 for STG (59%, 95% CI 49–68%) and SFG (24%, 95% CI 17–23%), while Mandalay had the highest percentage with ELISA OD ≥ 0.5 for TG (20%, 95% CI 13–29%).

### Seropositivity estimated by IFA

**Figure 3:**
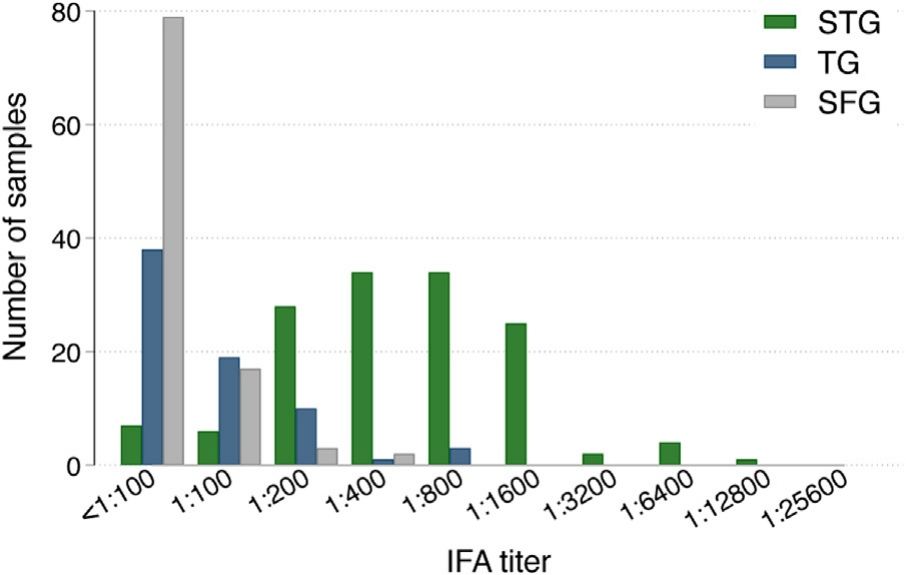
Immunofluorescence assay (IFA) titres of all samples with enzyme-linked immunosorbent assay optical density ≥0.5 for scrub typhus group (STG), typhus group (TG) and spotted fever group (SFG).

**Figure 4:**
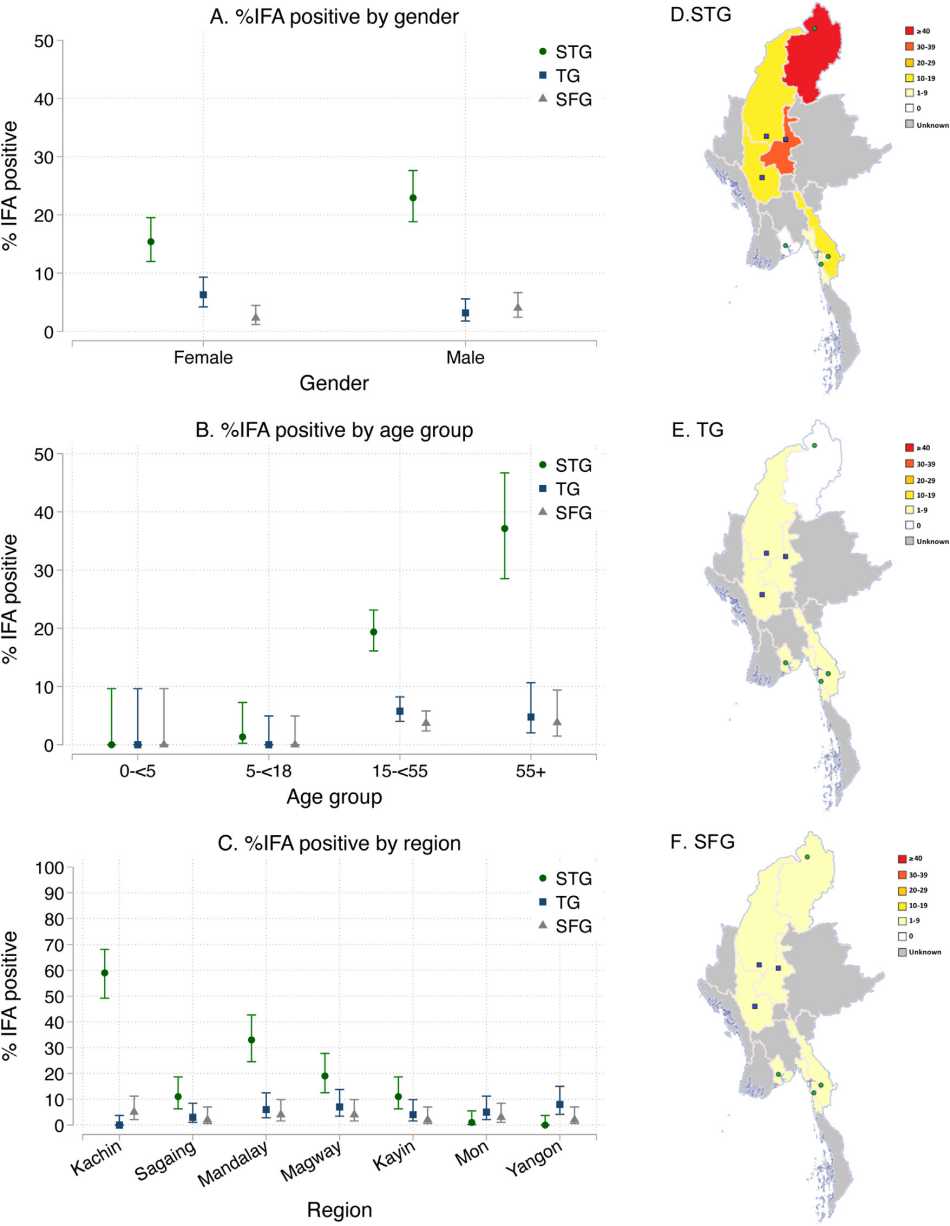
Percentages and 95 % confidence intervals of seropositive participants out of all participants displayed by gender (A), age group (B) and region (C) with maps (D, E, F) displaying the percentage in each region for scrub typhus group (STG), typhus group (TG) and spotted fever group (SFG), respectively. Seropositivity was defined as an immunofluorescence assay (IFA) titre ≥1:100 on samples with enzyme-linked immunosorbent assay (ELISA) optical density (OD) ≥ 0.5. All participants with ELISA OD < 0.5 were not tested with IFA, and were considered to be seronegative.

Of the 700 screened samples, 141, 71 and 101 had ELISA OD ≥ 0.5 and were therefore tested with IFA for STG, TG and SFG IgG titres, respectively, to confirm possible seropositivity. Of the 700 participants, 19.1% (95% CI 16.4–22.2%) were seropositive for STG, 4.7% (95% CI 3.4–6.5%) were seropositive for TG and 3.1% (95% CI 2.0–4.7%) were seropositive for SFG [Figure 3 and Table S3 (see online supplementary material)]. For all rickettsial infections, seropositivity was 0–1% in participants aged <18 years, with higher antibody levels in older age groups; this was particularly true for STG, with 37% of participants aged ≥55 years being seropositive (Figure 4). The highest seroprevalence of STG was found in Kachin (59%, 95% CI 49–68%), followed by Mandalay (33%, 95% CI 25–43%) and Magway (19%, 95% CI 13–28%), with several participants with evidence of seropositivity in Sagaing and Kayin (both 11%, 95% CI 6–19%), and almost none in Mon (1%, 95% CI 0–5%) and Yangon (0%, 95% CI 0–4%). Seroprevalence of TG and SFG was low in all regions (0–8% and 2–5%, respectively) (Figure 4). Two participants had an IFA titre ≥1:100 for both STG and TG, eight participants had an IFA titre ≥1:100 for STG and SFG, three participants had an IFA titre ≥1:100 for TG and SFG, and no participants had an IFA titre ≥1:100 for all three groups.

### Logistic regression seropositivity

**Figure 5:**
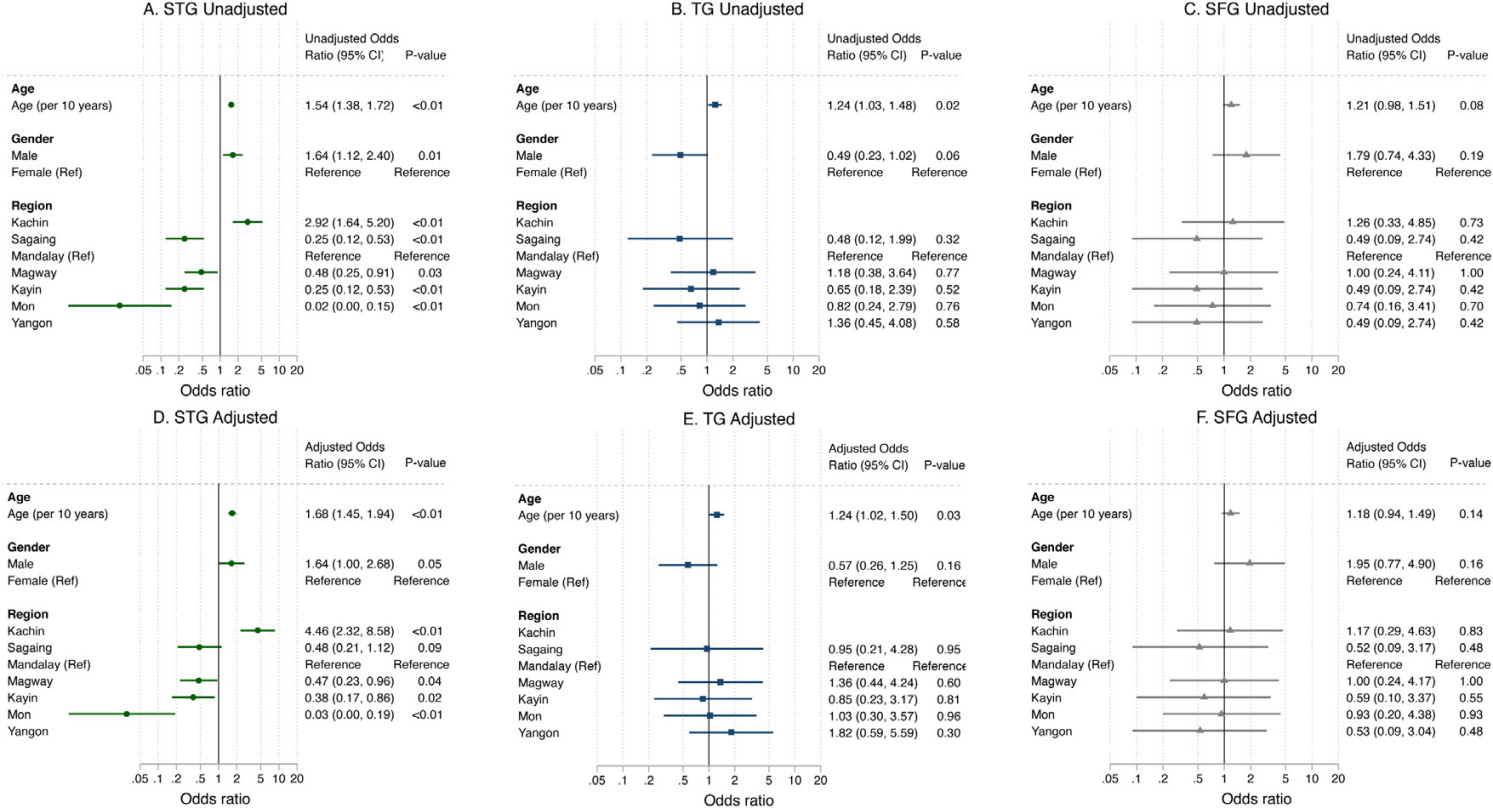
Unadjusted (A, B, C) and adjusted (D, E, F) odds ratios for seropositivity using logistic regression analysis. The variables that were entered were age per 10 years, gender (female as reference) and region (Mandalay as reference). Odds ratios with 95 % confidence intervals are displayed for scrub typhus group (STG), typhus group (TG) and spotted fever group (SFG). No participants were seropositive for STG in Yangon, and no participants were seropositive for TG in Kachin. Seropositivity was defined as immunofluorescence assay (IFA) titre ≥1:100 following enzyme-linked immunosorbent assay (ELISA) optical density (OD) ≥0.5. All participants with ELISA OD < 0.5 were not tested with IFA, and were considered to be seronegative.

Multivariable logistic regression was performed to estimate the association between different participant characteristics and the odds of seropositivity (Figure 5). Every 10-year increase in age was associated with higher odds of being seropositive for STG [adjusted odds ratio (aOR) 1.68, 95% CI 1.45–1.94) and TG (aOR 1.24, 95% CI 1.02–1.50), but not SFG (aOR 1.18, 95% CI 0.94–1.49). Males had higher odds of being seropositive for STG compared with females (aOR 1.64, 95% CI 1.00–2.68), but this was not the case for TG (aOR 0.57, 95% CI 0.26–1.25) or SFG (aOR 1.95, 95% CI 0.77–4.90). All regions were compared with Mandalay as the reference group. For STG seropositivity, aOR was 4.46 (95% CI 2.32–8.58) for Kachin, 0.03 (95% CI 0.00–0.19) for Mon and 0.47 (95% CI 0.23–0.96) for Magway compared with Mandalay. It was not possible to calculate the OR for STG seropositivity in Yangon or the OR for TG seropositivity in Kachin as none of the participants were seropositive. No significant differences in TG and SFG seropositivity were found between Mandalay and the other regions.

## Discussion

This study aimed to determine the seroprevalence of previous rickettsial infections in different regions of Myanmar. IFA results showed evidence of STG antibodies in almost 20% of participants, TG antibodies in 5% of participants and SFG antibodies in 3% of participants in seven different regions of Myanmar. The overall seroprevalence of STG in this study was similar to the median seroprevalence (22.2%) reported in a systematic review of scrub typhus in Asia (Bonell et al., 2017). Seroprevalence studies on rickettsial infections in other (Southeast) Asian countries have found differing prevalence rates; in Bhutan and Northeastern India, similar seroprevalence of STG and TG, but higher seroprevalence of SFG were found; in Bangladesh, much higher seroprevalence of TG was found; and in an indigenous Malaysian population, much higher seroprevalence of TG and SFG was found (Khan et al., 2016, Maude et al., 2014, Tappe et al., 2019, Tshokey et al., 2017). In healthy individuals in northern Vietnam, lower seroprevalence was found for STG, but similar seroprevalence for TG and SFG (Trung et al., 2017). The present finding of high seroprevalence of STG in Kachin was also reported in studies performed during the Second World War (Mackie, 1946, Sayen and Pond, 1946), and is reflected in the findings of recent Chinese studies on the spatio-temporal patterns of scrub typhus infections in mainland China which reported a high incidence cluster in Chinese regions bordering Kachin (Wu et al., 2016; Yue et al., 2019). Furthermore, a recent report on seropositivity and genotypic diversity in suspected cases of scrub typhus in Sagaing and Magway confirms the present results regarding the prevalence of STG in these regions (Win et al., 2020). The findings of these seroprevalence studies are difficult to compare due to different inclusion criteria; for example, many studies included adults alone, leading to higher seroprevalence than reported in the present study, and studies used a range of serological antibody tests with different cut-off points. There is a need for standardized testing using similar serological antibody tests, cut-offs and clear reporting of results to facilitate comparison of results. However, these studies do show that most rickettsial infections are still highly prevalent in Southeast Asia, with the present study providing the first comprehensive evidence of different types of rickettsial infections in different regions of Myanmar.

The difference in distribution of and exposure to vectors transmitting the different rickettsial infections could likely explain a large part of the variation of seroprevalence found between the different regions of Myanmar. Scrub typhus is most common in more rural areas with transitional habitats, such as along forest edges, shrubs and grasses, as these conditions favour chiggers and their rodent hosts (Chaisiri et al., 2017, Traub and Wisseman, 1974). Participants in the more rural area of Kachin in northern Myanmar had much higher odds of seropositivity compared with those in Mandalay, and a high number of participants with STG seropositivity compared with the urban slum area of Yangon, which had no participants that tested positive with the screening ELISA for STG antibodies. This finding is supported by a previous study on the causes of fever in primary care in Yangon, which found no evidence for scrub typhus as a cause of fever in several hundred patients (Althaus et al., 2020). Murine typhus is typically carried by fleas on rats and thrives in more urban areas (Aung et al., 2014). This was reflected in the finding that rural Kachin had no participants with TG seropositivity, but other less rural areas had a higher number of seropositive participants. The SFG rickettsiae tested for in this study are transmitted by ticks and are usually located in more rural areas (Aung et al., 2014), but no difference in seropositivity was found between regions in this study. It is not known if participants had moved between regions and had different levels of exposure to vectors. Future research could therefore focus on studying seroprevalence in all regions of Myanmar in acute infections, or in a longitudinal cohort trying to establish the effects of specific factors on rickettsial disease dynamics, such as (agricultural) land-use change, climate change and interactions with (domesticated) animals (Morand et al., 2020, Shah et al., 2019).

Increasing age was associated with higher odds of seropositivity and higher antibody levels for STG and TG, in agreement with previous studies (Maude et al., 2014, Richards et al., 1997). Older participants with higher antibody levels have likely had repeated exposure over time or more recent exposure to rickettsial infections, leading to higher baseline IgG antibody levels (Kim et al., 2010, Phakhounthong et al., 2020). IgG titres for rickettsial infections usually increase after the second week of illness, peak 1 month to several months after acute infection, and decline relatively rapidly after the first several months to years (Kim et al., 2010, Phakhounthong et al., 2020, Schmidt et al., 2019, Varghese et al., 2018). The relatively rapid decline in IgG antibodies over time indicates that the actual number of participants who had been infected with a rickettsial infection at any point in their life was likely to be higher than reported in this study, but these participants were no longer seropositive for IgG due to antibody decay.

A potential limitation of this study could be cross-reactivity between TG and SFG antibodies (La Scola and Raoult, 1997, Robinson et al., 2019). However, this study found that only three participants were seropositive for both TG and SFG, indicating that cross-reactivity between different rickettsial groups with IFA was unlikely. Another potential limitation of this study was that only a subset of the participants was tested with IFA after screening with ELISA, from which all participants with ELISA OD ≥ 0.5 were presumed to have an IFA titre <1:100. Initial screening with ELISA followed by IFA has been validated with high sensitivity (>95%) for both STG and TG (Elders et al., 2020, n.d.), but not yet for SFG. More research is needed to validate ELISA compared with IFA for SFG. Future research should also focus on characterizing locally circulating strains of rickettsial infections. This is particularly true for SFG as there is evidence of other species of SFG rickettsiae on the Thai–Myanmar border (Parola et al., 2003, Phongmany et al., 2006) which were not tested for in this study, leading to likely underestimation of the true burden of rickettsial infections.

In conclusion, this study showed the presence of all major groups of rickettsial infections in Myanmar, with increasing exposure with age, and particularly high prevalence of scrub typhus in northern and central Myanmar. The findings of this study show that rickettsial infections should be considered as possible causes of non-malarial febrile illnesses in Myanmar. This could be particularly relevant for the treatment of central nervous system infections (Dittrich et al., 2015). Future research should focus on improving serological and other diagnostic tests for rickettsial infections, studying the prevalence of rickettsial infections in all regions of Myanmar, characterizing locally circulating strains, and studying how disease dynamics are affected by factors such as land-use change, so that the disease burden of rickettsial infections can be properly estimated and addressed in Myanmar.

## Conflict of interest

None declared.

## Funding

This work was supported by the private donors of Medical Action Myanmar, and Wellcome Trust funding of MORU Tropical Health Network.

## Contributors

This study was conceptualized by PE, SB and EA. Methodology of data collection and analysis was set up by PE, AM, WYAH, NNT, YYH, SB and EA. Data collection was coordinated by MMMS, APP and KS, and performed by MMMS, APP, HNL, TKH, TTN, MMM, WMT, NNZ, WMK and HL. Laboratory analysis was performed and supervised by AT and SB. Data analysis and figure creation was undertaken by PE with technical support from AM. The manuscript was written by PE and EA. Funding acquisition and supervision were undertaken by FS, SB and EA. All authors reviewed the manuscript, added appropriate revisions, agreed to submission for publication, and approved the final version.

## Ethical approval

This study was approved by the University of Public Health Institutional Review Board, Yangon, Myanmar (UPH-IRB 2019/Research/22) and the Oxford Tropical Research Ethics Committee, Oxford, UK (OxTREC Reference 552−18).
