# Supplementary material for: Serological evidence indicates widespread distribution of rickettsioses in Myanmar
Source: Int J Infect Dis. 2021 Feb;103:494–501. doi: 10.1016/j.ijid.2020.12.013 (PMC7862081; doi:10.1016/j.ijid.2020.12.013)
Supplement: Supplementary file 1 [file mmc1.docx]

**Supplementary material - Rickettsioses in Myanmar**

**Supplementary Table 1.** Median ELISA Optical Density (percentile(p)25, p75) for for scrub typhus group (STG), typhus group (TG) and spotted fever group (SFG) IgG by participant demographic.

| **Demographic** | **Number of Samples** | **STG IgG** | **TG IgG** | **SFG IgG** |
| --- | --- | --- | --- | --- |
| **Gender** |  |  |  |  |
| male | 349 | 0.06 (0.02, 0.46) | 0.09 (0.06, 0.17) | 0.13 (0.04, 0.38) |
| female | 351 | 0.05 (0.02, 0.22) | 0.12 (0.08, 0.24) | 0.11 (0.04, 0.29) |
| **Age group, years** |  |  |  |  |
| 0-<5 | 36 | 0.01 (0.01, 0.03) | 0.07 (0.04, 0.10) | 0.02 (0.01, 0.13) |
| 5-<18 | 74 | 0.02 (0.01, 0.07) | 0.10 (0.08, 0.14) | 0.04 (0.02, 0.14) |
| 18-<55 | 485 | 0.06 (0.02, 0.36) | 0.11 (0.06, 0.18) | 0.13 (0.04, 0.33) |
| ≥55 | 105 | 0.26 (0.04, 0.83) | 0.17 (0.08, 0.45) | 0.21 (0.06, 0.47) |
| **Location (Region)** |  |  |  |  |
| Putao (Kachin) | 100 | 0.69 (0.16, 1.08) | 0.05 (0.04, 0.07) | 0.20 (0.04, 0.49) |
| Monywa (Sagaing) | 100 | 0.04 (0.01, 0.24) | 0.07 (0.03, 0.12) | 0.07 (0.02, 0.35) |
| Mandalay (Mandalay) | 100 | 0.27 (0.05, 0.79) | 0.09 (0.06, 0.38) | 0.12 (0.02, 0.31) |
| Magway (Magway) | 100 | 0.06 (0.03, 0.34) | 0.12 (0.08, 0.23) | 0.12 (0.03, 0.28) |
| Winka (Kayin) | 100 | 0.04 (0.02, 0.15) | 0.13 (0.09, 0.20) | 0.17 (0.05, 0.42) |
| Thanbyuzayat (Mon) | 100 | 0.03 (0.02, 0.06) | 0.14 (0.11, 0.28) | 0.10 (0.03, 0.31) |
| Yangon (Yangon) | 100 | 0.03 (0.01, 0.06) | 0.13 (0.10, 0.21) | 0.11 (0.05, 0.21) |
| **Total** | **700** | **0.05 (0.02, 0.34)** | **0.11 (0.07, 0.19)** | **0.13 (0.04, 0.32)** |

**Supplementary Table 2.** Percentage of samples positive using ELISA Optical Density ≥0.5 for rickettsial IgG as cut-off point with 95% confidence intervals for each gender, age group and region for scrub typhus group (STG), typhus group (TG) and spotted fever group (SFG)..

| **Demographic** | **Number of Samples** | **STG IgG** | | **TG IgG** | | **SFG IgG** | |
| --- | --- | --- | --- | --- | --- | --- | --- |
|  |  | **% pos** | **95% CI** | **% pos** | **95% CI** | **% pos** | **95% CI** |
| **Gender** |  |  |  |  |  |  |  |
| male | **349** | **24** | **20-29** | 7 | 5-11 | 18 | **15-23** |
| female | 351 | **17** | **13-21** | 13 | 10-17 | 11 | **8-14** |
| **Age group, years** |  |  |  |  |  |  |  |
| 0-<5 | 36 | **0** | **0-10** | 3 | 1-14 | 6 | **2-18** |
| 5-<18 | 74 | **3** | **1-10** | 3 | 1-9 | 3 | **1-9** |
| 18-<55 | 485 | **20** | **17-24** | 9 | 7-12 | 15 | **12-18** |
| ≥55 | 105 | **39** | **30-49** | 22 | 15-31 | 24 | **17-33** |
| **Location (Region)** |  |  |  |  |  |  |  |
| Putao (Kachin) | 100 | 59 | 49-68 | 2 | 0-7 | 24 | 17-33 |
| Monywa (Sagaing) | 100 | 11 | 6-19 | 4 | 2-10 | 15 | 9-23 |
| Mandalay (Mandalay) | 100 | 35 | 26-45 | 20 | 13-29 | 12 | 7-20 |
| Magway (Magway) | 100 | 20 | 13-29 | 15 | 9-23 | 7 | 3-14 |
| Winka (Kayin) | 100 | 14 | 9-22 | 4 | 2-10 | 22 | 15-31 |
| Thanbyuzayat (Mon) | 100 | 2 | 0-7 | 13 | 8-21 | 13 | 8-21 |
| Yangon (Yangon) | 100 | 0 | 0-4 | 13 | 8-21 | 8 | 4-15 |
| **Total** | **700** | **20.1** | **17.3-23.3** | **10.1** | **8.1-12.6** | **14.4** | **12.0-17.2** |

**Pos=positive; CI=confidence intervals**

**Supplementary Table 3.** Number of samples tested with IFA after screening with ELISA and considered seropositive out of total number of participants for gender, age group and region. IFA samples were considered seropositive with a titre≥1:100 for scrub typhus group (STG), typhus group (TG) and spotted fever group (SFG).

| **Demographic** | **N** | **STG IgG IFA** | | | **TG IgG IFA** | | | **SFG IgG IFA** | | |
| --- | --- | --- | --- | --- | --- | --- | --- | --- | --- | --- |
|  |  | **No of IFA** | **% pos of total N** | **95% CI** | **No of IFA** | **% pos of total N** | **95% CI** | **No of IFA** | **% pos of total N** | **95% CI** |
| **Gender** |  |  |  |  |  |  |  |  |  |  |
| male | 349 | 83 | 23 | 19-28 | 26 | 3 | 2-6 | 64 | 4 | 2-7 |
| female | 351 | 58 | 15 | 12-20 | 45 | 6 | 4-9 | 37 | 2 | 1-4 |
| **Age group, years** | | | | | | | | | | |
| 0-<5 | 36 | 0 | 0 | 0-10 | 1 | 0 | 0-10 | 2 | 0 | 0-10 |
| 5-<18 | 74 | 2 | 1 | 0-7 | 2 | 0 | 0-5 | 2 | 0 | 0-5 |
| 18-<55 | 485 | 98 | 20 | 16-23 | 45 | 6 | 4-8 | 72 | 4 | 2-6 |
| ≥55 | 105 | 41 | 37 | 29-47 | 23 | 5 | 2-11 | 25 | 4 | 1-9 |
| **Location (Region)** | | | | | | | | | | |
| Putao (Kachin | 100 | 59 | 59 | 49-68 | 2 | 0 | 0-4 | 24 | 5 | 2-11 |
| Monywa (Sagaing) | 100 | 11 | 11 | 6-19 | 4 | 3 | 1-8 | 15 | 2 | 1-7 |
| Mandalay (Mandalay) | 100 | 35 | 33 | 25-43 | 20 | 6 | 3-12 | 12 | 4 | 2-10 |
| Magway (Magway) | 100 | 20 | 19 | 13-28 | 15 | 7 | 3-14 | 7 | 4 | 2-12 |
| Winka (Kayin) | 100 | 14 | 11 | 6-19 | 4 | 4 | 2-10 | 22 | 2 | 1-7 |
| Thanbyuzayat (Mon) | 100 | 2 | 1 | 0-5 | 13 | 5 | 2-11 | 13 | 3 | 1-8 |
| Yangon (Yangon) | 100 | 0 | 0 | 0-4 | 13 | 8 | 4-15 | 8 | 3 | 1-7 |
| **Total** | **700** | **141** | **19.1** | **16.4-22.2** | **71** | **4.7** | **3.4-6.5** | **101** | **3.1** | **2.0-4.7** |

N=number of participants; no=number; pos=positive; CI=confidence intervals
